# Binding of TS1, an anti-keratin 8 antibody, in small-cell lung cancer after ^177^Lu-DOTA-Tyr^3^-octreotate treatment: a histological study in xenografted mice

**DOI:** 10.1186/2191-219X-1-19

**Published:** 2011-08-26

**Authors:** Ann Erlandsson, Eva Forssell-Aronsson, Tomas Seidal, Peter Bernhardt

**Affiliations:** 1 Department of Chemistry and Biomedical Sciences, Karlstad University, 651 88 Karlstad, Sweden; 2 Department of Radiation Physics, Sahlgrenska Academy, University of Gothenburg, Sahlgrenska University Hospital, SE-413 45 Göteborg, Sweden

**Keywords:** ^177^Lu-DOTA-Tyr^3^-octreotate, somatostatin receptor subtype 2 (SSTR2), small-cell lung cancer (SCLC), keratin 8 (K8)

## Abstract

**Background:**

Small-cell lung carcinoma (SCLC) is an aggressive malignancy characterised by an early relapse, a tendency towards drug resistance, and a high incidence of metastasis. SCLC cells are of neuroendocrine origin and express high levels of somatostatin receptors; therefore, future treatment might involve targeting tumours with radiolabelled somatostatin analogues. This therapy induces abundant necrotic patches that contain exposed keratins; thus, keratin 8, which is one of the most abundant cytoskeletal proteins may represent an interesting secondary target for SCLC. This study aimed to investigate the effects of^177^Lu-DOTA-Tyr^3^-octerotate and the binding of the monoclonal anti-keratin 8 antibody, TS1, *in vitro *in treated SCLC- and midgut-xenografted mouse models.

**Methods:**

NCI-H69- and GOT1-xenotransplanted mice were treated with three doses of 30 MBq^177^Lu-DOTA-Tyr^3^-octreotate administered 24 h apart. Mice xenotransplanted with NCI-H69 were sacrificed 1, 5, 12, 20 and 150 days post-injection or when the tumour had regrown to its original size. GOT1-xenotransplanted mice were sacrificed 3 days post-injection. Immunohistochemistry was performed to evaluate TS1 staining in tumours and in seven human biopsies of primary SCLC from pulmonary bronchi. Central cell density and nucleus size were determined in NCI-H69 sections.

**Results:**

Twelve days after^177^Lu-DOTA-Tyr^3^-octerotate treatment, the SCLC xenograft response was extensive. Twenty days after treatment, one of three analysed tumours displayed complete remission. The other two tumours showed 1/4 the cell density of untreated controls and cell nuclei were about three times larger than those of untreated controls. At 150 days after treatment, one of four mice exhibited complete remission. Treated tumours displayed increased TS1 antibody accumulation and high TS1 binding in necrotic patches. All seven human SCLC biopsies displayed necrotic areas with TS1 staining.

**Conclusions:**

Radiation treatment with three injections of 30 MBq^177^Lu-DOTA-Tyr^3^-octreotate had pronounced effects on tumour cell density and cell nuclei, which indicated mitotic catastrophe. Despite these anti-tumour effects, two of three SCLC tumours recurred. Further studies should investigate the nature of tumour cell survival and develop more effective treatments. High TS1 accumulation in tumour sections *in vitro *after^177^Lu-DOTA-Tyr^3^-octerotate treatment indicated that TS1 might represent a promising secondary therapeutic strategy.

## Background

Small-cell lung carcinoma (SCLC) comprises about 15-20% of all diagnosed lung cancers. The prognosis of this disease is often poor; distant metastases are typically observed at the time of diagnosis. New improved treatment modalities are urgently needed and have been intensively discussed [1-3]. SCLC is characterised by small- to medium- sized, tightly packed, highly mitotic cells that generate prominent necrotic areas [4]. The origin of SCLC is neuroendocrine; the tumour cells express neuroendocrine markers, somatostatin receptors, and keratin 8, 18, and 19 [5-7]. In an effort to understand the nature of these tumours, previous studies have investigated the presence of cells with stem cell characteristics [8-10], the consequences of the lack of wild-type p53 [11,12], and the over expression of Bcl-2 [13,14]. About 80-100% of all SCLC cells express somatostatin receptor subtype 2 (SSTR2). Somatostatin receptor scintigraphy can be used to visualise primary tumours and metastases [15,16]. Radiolabelled somatostatin analogues have been tested as a single therapy for SCLC, but this has not been as promising as predicted, based on preclinical study results [17-19]. However, the number of clinical studies and the number of patients in those studies have been relatively limited and the treatment protocol was not optimised for those patients [20,21]. In contrast, patients with gastro-entero-pancreatic tumours have been treated successfully with somatostatin analogues [22,23]; furthermore, exogenous gene transfer of the SSTR2 gene into SSTR-negative tumours has enabled treatment with somatostatin analogues [24]. Previous animal studies have shown that the somatostatin analogue, octreotate, labelled with^177^Lu (^177^Lu-DOTA-Tyr^3^-octreotate) might be a promising treatment. Dosimetric studies revealed that the therapeutic radionuclide,^177^Lu, had physical properties beneficial for therapy [25,26]. Because^177^Lu emits medium-energy electrons, it is suitable for treating a wide range of tumour sizes. Its long physical half-life of 6.7 days and its higher retention in tumours compared to normal tissues provides an optimal ratio of tumour to normal tissue dose absorption. In a study by Schmitt et al., nude mice that bore tumours of the human SCLC cell line, NCI-H69, were used as a preclinical model. The results demonstrated that single doses of 45-120 MBq of^177^Lu-DOTA-Tyr^3^-octreotate caused extensive tumour regression during the first 1-3 weeks after treatment [18]. In the same study, two 45-MBq fractions of^177^Lu-DOTA-Tyr^3^-octreotate given 48 h apart caused more extensive tumour regression. The tumours continued to decline over the entire study period (34 days) [18]. However, despite these promising results with fractionation protocols, a later study by Kolby et al. demonstrated that the SSTR2 receptors were saturated when doses above 30 MBq were given in one fraction [27]. Therefore, a more frequent fractionation pattern with lower doses might be more effective in a therapeutic setting. Another study demonstrated that^177^Lu-DOTA-Tyr^3^-octreotate treatment caused an up-regulation in somatostatin receptor mRNA expression. That result supported the idea that fractionated doses might induce receptor expression and, consequently, increase the uptake of^177^Lu-DOTA-Tyr^3^-octreotate [28,29]. However, new combinations of therapies should be evaluated to achieve more effective treatments for SCLC.

We hypothesised that somatostatin therapy combined with an anti-keratin antibody might comprise an effective SCLC treatment. The^131^I-radiolabelled monoclonal antibody, thrombospondin-1 (TS1) [30], which binds human keratin 8 (K8), has been successfully used in experimental radiotherapy for HeLa cell tumours [31,32]. K8 is a member of the intermediate filament family, constitutes an important part of the cell cytoskeleton, and is involved in many dynamic cellular processes, like migration, invasion, and metastasis [33]. It has also been demonstrated that the carboxyl-terminal of K8 can function as a plasminogen receptor [34]. In addition, some cell lines have highly tumourigenic clones with increased expression of K8 [35]. The antibody TS1 binds with high affinity to a unique epitope in K8 between amino acids 343 and 357; the abundance of this epitope is very high in necrotic regions, due to the low solubility of the K8 antigen and the high concentration of the antigen in tumour cells [36]. Because SCLC tumours have extensive necrotic regions [4], radiolabelled TS1 antibody accumulation could be sufficient for effective radiation of the tumours. Targeted radiotherapy with^177^Lu-DOTA-Tyr^3^-octreotate might further increase the cytoplasmic release of K8 and thereby increase the accumulation of radiolabelled TS1 antibodies in SCLC tumours.

The main purpose of this study was to investigate the binding of TS1 in small-cell lung cancer xenografts after^177^Lu-DOTA-Tyr^3^-octreotate treatment. We analysed changes in tumour cell density, nuclear size, and binding of TS1, Ki67, and Bcl-2 at different time points. We also confirmed TS1 binding in a^177^Lu-DOTA-Tyr^3^-octreotate-treated xenografts of the midgut carcinoid cell line GOT1 and in tumour biopsies from seven patients with small-cell carcinoma in pulmonary bronchi.

## Materials and methods

### Animals and cell line

Four-week-old male BALB/c mice (Bommice; weight, 25 ± 0.5 g) underwent subcutaneous implantations in the neck with 2 × 10^7 ^cells of the human SCLC cell line, NCI-H69 (ATCC HTB-119; American Type Culture Collection, Manassas, VA, USA) or the midgut carcinoid cell line GOT1 [37]. Tumours were allowed to grow for 4 weeks to a diameter of 10-15 mm. These experiments were approved by the Ethical Committee for Animal Experiments, University of Gothenburg. The animals were given autoclaved food and drinking water *ad libitum*. Tumour growth, animal condition, and body weight were determined at regular intervals. The animals were killed when they lost > 10% of their original body weight or when the tumour reached a diameter > 20 mm.

### Patient material

This study included biopsies from seven cases of human small-cell carcinoma in pulmonary bronchi that had not been treated with radiolabelled somatostatin analogues. Biopsies were fixed in formalin, embedded in paraffin, and cut into 4-μm-thick sections. Sections from all seven tumours were stained immunohistochemically with TS1 and sections from two tumours were analysed with anti-SSTR2. The tissue material was decoded before processing to remove links that could identify patients.

### Radiopharmaceuticals

DOTA-Tyr^3^-octreotate (synthesised at the Institute of Nuclear Medicine, University Hospital, Basel, Switzerland) was dissolved in a 0.05 M HCL solution that contained 126 mg/ml sodium ascorbate and 25 mg/ml gentisic acid (2,5-dihydroxybenzoic acid) [17,18].

^177^LuCl_3 _with 740 MBq/μg maximal specific activity was obtained from Perkin Elmer (St. Louis, MO, USA).^177^LuCl_3 _was diluted to 4.2-5.3 MBq/μl and incubated with the DOTA-Tyr^3^-octreotate solution for 30 min at 80°C. This resulted in DOTA-Tyr^3^-octreotate with a specific activity of 30 MBq/μg. With instant thin layer chromatography (ITLC-SG, Gelman, Ann Arbor, MI, USA) performed as described previously [17,18], we determined that > 98%^177^Lu was bound to peptide.

### Therapeutic study

Twenty SCLC-xenografted mice were injected in a lateral tail vein with three fractions of 30 MBq^177^Lu-DOTA-Tyr^3^-octreotate in 0.1-0.2 mL isotonic saline given 24 h apart. One control group of the SCLC-xenografted mice received no treatment (*n *= 5); the mice in this group were killed after 20 days. The^177^Lu-DOTA-Tyr^3^-octreotate-treated SCLC-xenografted mice were divided into five groups that were sacrificed at 1 day (*n *= 5), 5 days (*n *= 5), 12 days (*n *= 4), 20 days (*n *= 3), and 150 days after treatment or until the tumour had regrown to the original size (*n *= 4). The GOT1-xenografted mice were divided into two groups untreated (*n *= 3) and 3 days (*n *= 3) of treatment. Tumour tissues were dissected, fixed in buffered formalin for 4 to 24 h, and embedded in paraffin. The tumours from each group were cut in serial sections, 4 μm-thick, and subjected to haematoxylin/eosin (H&E) staining, immunohistochemistry, and microscopy to determine cell numbers (tumour and mouse cells) and cell nucleus size.

### Dosimetry

The dosimetry calculations were based on the pharmacokinetics determined previously in NCI-H69 and GOT1 bearing nude mice [19,38]. In both tumours, instant uptake was assumed; for NCI-H69 and GOT1, the instant uptake rates were 5.8% IA/g and 14% IA/g, respectively. For H69 tumours, activity concentrations were fit with a declining monoexponential; the effective half-life was determined to 5.8 days. We assumed that the emitted electrons were totally absorbed in tumour tissues. We neglected the small photon dose absorbed in tumours. The different fractions were assumed to have similar biokinetics, and the cumulative activity was determined by integrating the monoexponential function up to the time point of sacrifice. We determined the mean absorbed dose by multiplying the cumulative activity by the emitted electron energy per decay; we expressed the results in gray units. A similar calculation was used for the mean absorbed dose in GOT1 tumours, except we could not use a declining monoexponential fit, because the activity concentration increased during therapy [38]. Instead, we fit the initial 7-day increase in concentration with a linear model; then, we fit the concentration over the remaining time with a declining monoexponential [38].

### Antibodies for immunohistochemistry

Tumour sections from control mice (no treatment) and from mice treated with^177^Lu-DOTA-Tyr^3^-octreotate were stained with the following antibodies: monoclonal mouse anti-human-Ki67 (Dako Glostrup, Denmark), monoclonal mouse anti-human-Bcl-2 oncoprotein (Dako, Glostrup, Denmark), monoclonal rabbit anti-somatostatin receptor subtype 2 (anti-SSTR2), HPA007264 (Sigma-Aldrich Corp., St. Louis, MO, USA), monoclonal mouse anti-human wild type-p53 (Dako Glostrup, Denmark), monoclonal mouse anti-human CD133/1 (Miltenyi Biotec, Bergisch Gladbach, Germany), monoclonal anti-human thyroid transcription factor (TTF-1) (Dako Glostrup, Denmark), polyclonal anti-human synaptophysin (Dako Glostrup, Denmark), and polyclonal anti-human chromogranin-A (Dako Glostrup, Denmark).

Hybridoma cell line that produced the monoclonal TS1 antibody, which reacted with keratin 8 (K8), was cultured as described previously [39]. The TS1 antibody was purified from cell culture media with a Protein G column (Amersham Pharmacia Biotech AB, Uppsala, Sweden) and eluted with 0.1 M glycine/HCl buffer, pH 2.3. Fractions were neutralised and stored at -20°C until use.

### Haematoxylin/eosin staining and immunohistochemistry

H&E staining was performed on a few selected slides from each group to study the morphology of the cells and determine the presence of necrosis, fibrosis, and apoptosis. Necrosis was defined as an area stained with haematoxylin that originated from proteins released from the cytoplasm of dead cells. Fibrosis was defined as the presence of proliferating fibroblast-like cells, often with an apoptotic appearance. Apoptotic cells were defined as cells with shrunken cytoplasms and nuclear chromatin condensation or intense DNA staining in condensed pyknotic nuclei.

The epitope retrieval procedure for commercial antibodies was performed as described by the manufacturer. For TS1, we performed heat-induced epitope retrieval in 10 mM Tris/10 mM EDTA-buffer, pH 9.0. For immunohistochemical staining, we used 5 μg/ml TS1 and the dilution recommended by the manufacturers for the commercial antibodies. A minimum of one section from three to five tumours from each group was analysed immunohistochemically with TS1, anti-Ki67, anti-Bcl-2. Sections were also analysed with each antibody, as follows: anti-somatostatin receptor subtype 2 antibody, one to three sections from each group; anti-human-p53, three sections to confirm negativity; anti-human CD133/1, one to two sections from each group; anti-human TTF-1, three sections to confirm negativity; anti-synaptophysin, five sections; and anti-chromogranin-A, five sections. Appropriate positive controls were used for all antibodies. The immunohistochemistry was performed with the standard method of horseradish peroxidise and diamonobenzidine (DAB) in a Dako autostainer with the EnVision standard reagents. After the immunostaining, the nuclei were counterstained with Mayer's haematoxylin, dehydrated, and mounted.

### Microscopic examination and calculation of the effect of different treatment periods

To evaluate the overall effect of^177^Lu-DOTA-Tyr^3^-octreotate in the SCLC xenograft, the ratio of tumour *versus *mouse cells was calculated and the sizes of cell nuclei were measured in a total of one to two sections from each of two to five tumours in the different treatment groups. A grid with 130 cross-points was placed over an area of 200 × 154 μm in the centre of each section. Tumour cells were defined as cells that stained positively for Ki67 or Bcl-2, cells with abnormal, angular, or polymorphic morphologies, or cells with a hyperchromatic nucleus and reduced cytoplasm. Those located within the cross-points of the grid were counted. Normal mouse cells were defined as cells that did not stain for Ki67 or Bcl-2 and had morphologies of normal mouse fibroblasts, adipose, and lymphoid cells.

In ten tumour cells, we measured the size of the nucleus, or the area with nuclear chromatin, in the cells in the centre of the section. We selected one to three sections from two to four tumours of each group, and systematically measured nuclei within the cross-points of the 200 × 154 μm grid, starting at the top left corner and continuing to the right bottom corner of the grid. The nucleus diameter measurement was performed in two directions and the value was expressed as the mean value and standard deviation. We used a Zeiss AXIO imager A1 microscope equipped with a Zeiss Axiocam. The calculations for the positively and negatively stained cells and for the nucleus size were confirmed with the AXIO Vision programme, release 4.7 (data not shown).

### Digital image analysis

The K8 and Ki67 expression levels in treated and untreated NCI-H69 SCLC and GOT 1 midgut tumour xenograft sections were quantified by analysing DAB staining with the ImageJ programme developed by the National Institute of Health [40]. The immunoRatio plugin of the programme measured the DAB (brown) and nuclei haematoxylin (blue) stains with an image resolution of 6.24 pixels/μm. Areas of 320 × 250 μm were analysed with the same light filter settings for each section. The imaged areas with tumour cells were randomly chosen, but areas of necrosis larger than 50 × 50 μm and mouse cells only were avoided. We analysed a total of 145 images from five, untreated SCLC tumour xenografts; 37 images from three, 1-day-treated, SCLC tumours; 74 images from four, 5-day-treated, SCLC tumours; 75 images from three, 12-day-treated, SCLC tumours; 33 images from two, 20-day-treated, SCLC tumours; and 57 images from three, regrown SCLC tumours. For the GOT 1 tumour xenografts, we analysed 63 images from three, untreated tumours and 83 images from four, 3-day-treated tumours. Between 10 and 40 sections from each group of SCLC and GOT1 xenografts were stained with Ki67 and similarly analysed. The mean percentages and standard deviations were calculated for K8 and Ki67 DAB stains. The results were compared with two-sided Students *t *tests and the significance level was set at *P *< 0.05.

Within each of the seven biopsies, two to nine 320 × 250 μm areas with tumour cells, necrotic tissue, and high TS1 staining were captured at a ×40 magnification and a resolution of 6.24 pixels/μm. These images were analysed with ImageJ plugin ImmunoRatio to calculate the percentage of DAB staining.

## Results

### Dosimetry

The injection of three times 30 MBq resulted in different mean absorbed doses to the H69 tumour tissue. For the mice sacrificed 1, 5, 12, 20, or 150 days after last injection the mean absorbed dose was estimated to be 13, 32, 51, 59, and 64 Gy, respectively. The mean absorbed dose to the GOT1 tumours was estimated to be 27 Gy.

### Haematoxylin/eosin staining

Histologically, the untreated control, 1-day-treated and regrown tumours showed the classical SCLC cell morphology with abnormal, angular, polymorphic, hyperchromatic nuclei, and scant cytoplasm. Large necrotic regions were present in the untreated, 1- and 5-day-treated tumours. After 12 and 20 days of treatment, the larger necrotic regions were replaced by fibrotic tissue to a large extent. H&E staining showed that the control tumours and regrown tumours were similar, but the regrown tumours had slightly larger areas of fibrosis. Compared to the untreated controls, treated tumours, particularly after 5 days, had higher numbers of small (50 × 50 μm) necrotic regions. Relatively few cells exhibited an apoptotic appearance with a shrunken cytoplasm and intensely stained DNA in condensed pyknotic nuclei. In tumours treated for 5 days, a slightly higher number of apoptotic cells with intense hematoxylin staining were observed between the necrotic areas and viable cells.

### Immunohistochemistry

**Figure 1:**
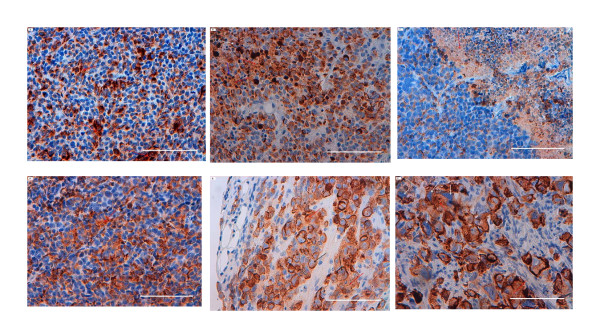
**^177^Lu-DOTA-Tyr^3^-octreotate treatment effects on tumour cells visualised by staining with the anti-K8 antibody, TS1**. (**A**) Untreated, midgut GOT1 xenograft; (**B**) Midgut GOT1 xenograft after 3 days of treatment; small necrotic areas are evident. (**C**) Untreated, SCLC NCI-H69 xenograft with viable, apoptotic, and necrotic cells; (**D**) SCLC NCI-H69 xenograft after 1 day of treatment; (**E**) SCLC NCI-H69 xenograft after 5 days of treatment; small necrotic areas are evident; and (**F**) SCLC NCI-H69 xenograft after 12 days of treatment. Red arrows indicate small necrotic areas, blue arrows point to apoptotic cells, and white or black arrows point to cells with multiple nuclei and micronuclei. Scale bar 100 μm.

**Figure 2:**
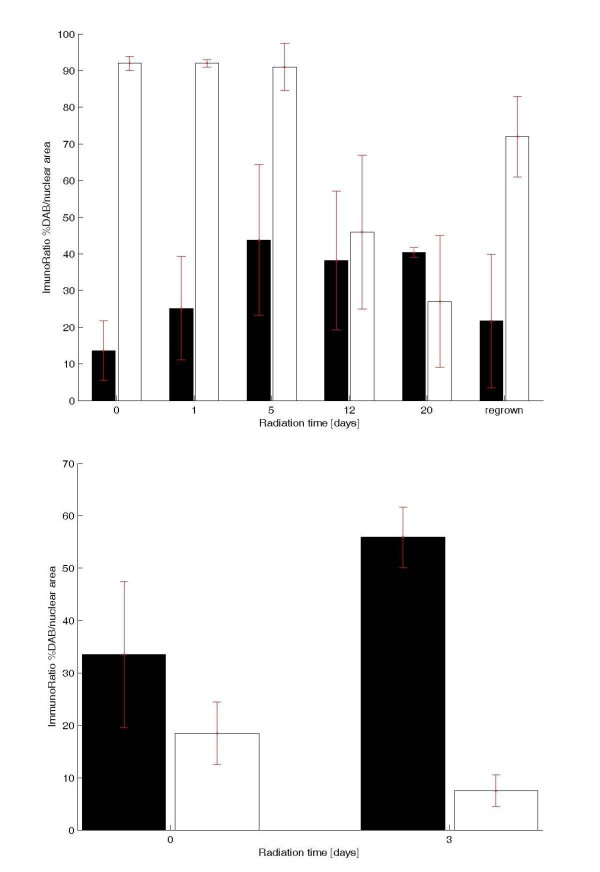
**Radiation effects on TS1 and Ki67 DAB-positive immunostaining**. (**a**) Radiation effects on TS1 and Ki67 DAB-positive immunostaining in the SCLC NCI-H69 xenograft. The *X*-axis shows the numbers of days treated with^177^Lu-DOTA-Tyr^3^-octreotate. The *Y*-axis shows the percentage (%) mean value and standard deviation of DAB-positive immunostaining for TS1 and Ki67. Bars that are close together represent results from different sections of the same tumour group. TS1 (black bars), Ki67 (white bars). The 5- and 12-days treatment differ significantly from the untreated, *p *< 0.05, 5-days *n *= 4 and 12-days *n *= 3. (**b**) Radiation effects on TS1 and Ki67 DAB-positive immunostaining in the midgut GOT1 xenograft. The *X*-axis shows the numbers of days treated with^177^Lu-DOTA-Tyr^3^-octreotate. The *Y*-axis shows the percentage (%) mean value and standard deviation of DAB-positive immunostaining for TS1 and Ki67. Bars that are close together represent results from different sections of the same tumour group. TS1 (black bars), Ki67 (white bars). Three-days treatment differ significantly from the untreated, *p *< 0.05, *n *= 4.

**Figure 3:**
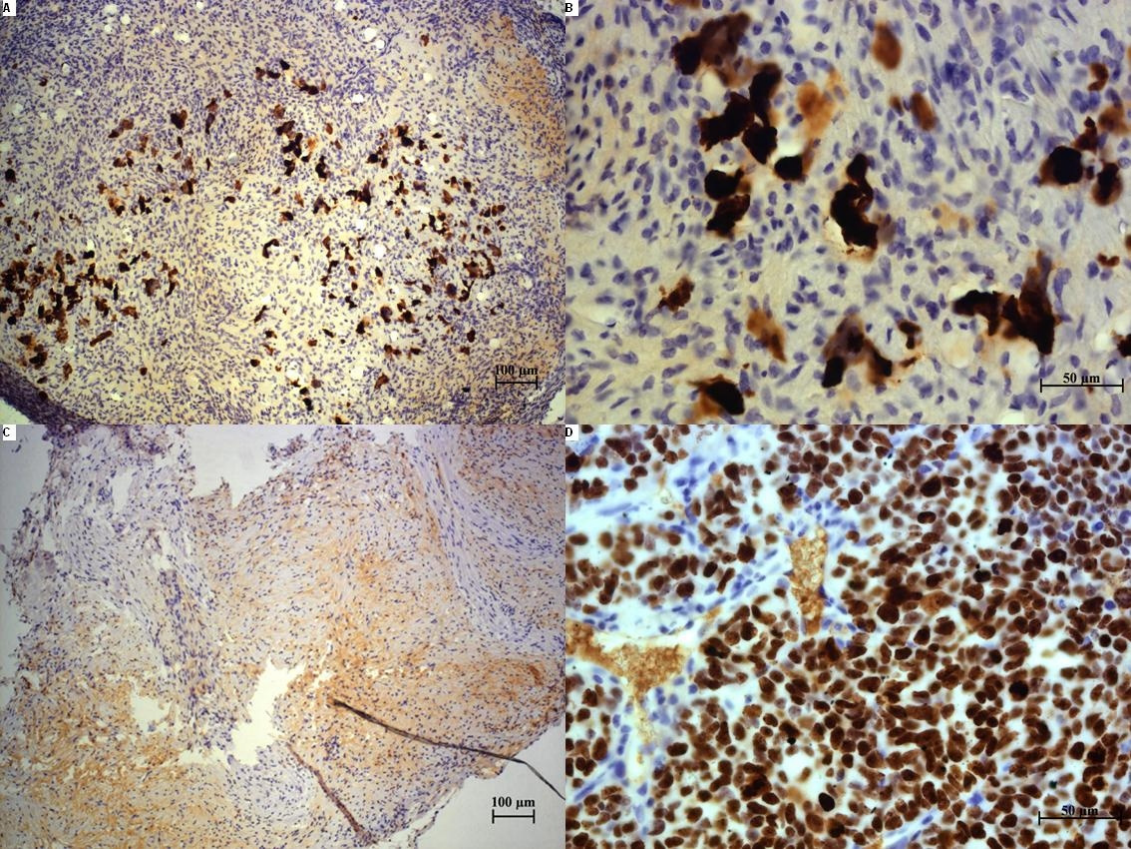
**Histological sections of treated and untreated SCLC tumours labelled with Ki67**. (**A**, **B**) Different magnifications of the same tumour after 20 days of treatment; (**C**) a Ki67-negative tumour after 20 days of treatment; (**D**) a representative untreated tumour labelled with Ki67.

**Figure 4:**
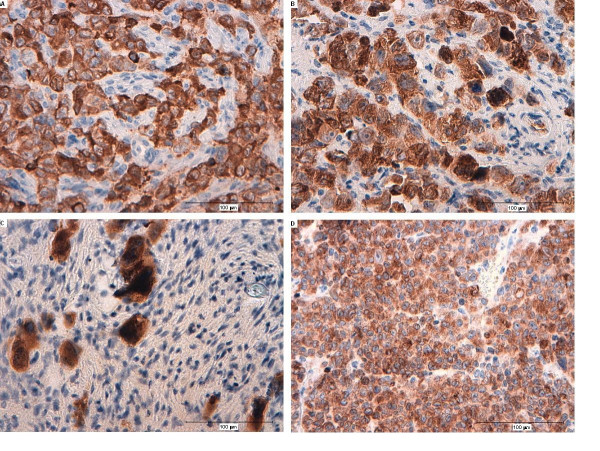
**Histological sections of treated SCLC tumours labelled with Bcl-2**. (**A**) A tumour after 5 days of treatment; (**B**) a tumour after 12 days of treatment; (**C**) a tumour after 20 days of treatment; Bcl-2 localisation is mainly in the nuclei; (**D**) a representative tumour that regrow after treatment; Bcl-2 localisation is mainly cytoplasmic.

The tumour cells from the xenograft of the human SCLC cell line NCI-H69 and the human cell line GOT1 stained positively with specific binding for TS1, anti-Ki67, and anti-Bcl-2. An increase in the intensity of the TS1, anti-Ki67, and anti-Bcl-2 staining per cell was also observed in tumour cells treated with^177^Lu-DOTA-Tyr^3^-octreotate (Figures 1, 2, 3, and 4).

All tumour cells in the SCLC xenograft were negative for the anti-CD-133/1, and as expected negative for anti-p53 and anti-TTF-1. The sections of the xenograft also stained positively, with some unspecific binding, for anti-SSTR2, anti-synaptophysin, and anti-chromogranin-A.

TS1 exhibited a distinct cytoplasmic pattern of staining in viable tumour cells, but also demonstrated binding to necrotic regions and pyknotic/apoptotic tumour cells (Figure 1). The staining of necrotic regions was high in both large and small areas, with some variation in staining intensity (Figure 1). The percentage of DAB staining (mean value and standard deviation) in each tumour was calculated with imageJ ImmunoRatio and presented in Table 1. Despite high variation in staining intensities between different tumours within the same group a significant increase could be seen between the untreated group and the 5- and 12-days-treated groups. Untreated SCLC NCI-H69 tumours showed 13% K8 expression, and this increased significantly to 44% after 5 days (*p *< 0.05; *n *= 4) and 38% after 12 days (*p *< 0.05; *n *= 3). After 20 days of treatment, the mean value in the two analysed tumours was 40% (Figure 2a). In GOT1 xenografts, untreated tumours showed 29% K8 expression, and this increased significantly to 58% after 3 days of treatment (*p *< 0.05; *n *= 3; Figure 2b).

**Table 1:** Level of TS1 staining in the SCLC NCI-H69 and GOT1 midgut xenografts

Tumour	Treatment	Images	Mean value	Standard deviation
1	Untreated SCLC	29	27.8	15.3
2		19	8.3	6.2
3		33	8.5	6.2
4		23	11.5	6.3
5		41	12.0	7.8
6	1-day SCLC	17	39.0	7.4
7		6	10.8	4.4
8		14	25.8	6.7
9	5-day SCLC	20	60.8	5.7
10		19	62.4	9.8
11		18	27.7	4.6
12		17	24.4	6.1
13	12-day SCLC	40	58.1	10.0
14		13	20.4	3.0
15		22	36.2	9.8
16	20-day SCLC	17	41.4	17.9
17		16	39.4	15.7
18	Regrown SCLC	18	20.3	13.0
19		15	40.7	10.3
20		24	4.2	2.5
21	Untreated midgut	18	40.3	10.5
22		13	42.7	14.1
23		32	17.6	15.3
24	3-day midgut	21	49.3	10.7
25		35	60.8	10.6
26		14	52.7	17.3
27		13	60.7	9.5

The table illustrates the number of images analysed, mean value, and standard deviation per analysed tumour. The percentage (%) DAB-positive immunostaining for TS1 in the tumour xenografts was determined by use of ImageJ ImmunoRatio.

Ki67, a marker for proliferation, is present in the nucleus in all active phases of the cell cycle, but absent in resting G_0 _cells. Cells with classical SCLC cell morphology in the untreated, one, five and regrown tumours exhibited 70-90% Ki67 staining, and after 12 and 20 days, the staining was reduced to 45% and 25% respectively (Figure 2). The Ki67 staining in the midgut cell line that was about 20% in the untreated tumour and was reduced to 10% in the 3-day-treated tumours (Figure 2a,b). Sections were interpreted as tumour cell negative when cells lacked the SCLC cell morphology and stained negative for Ki67 (Figure 3).

Bcl-2 is an integral membrane protein expressed in the endoplasmic reticulum (ER), the outer membranes of mitochondria, and in the nuclear envelope. In this study, Bcl-2 expression was located in the tumour cell cytoplasm (ER and/or mitochondria) of untreated tumours. During treatment with^177^Lu-DOTA-Tyr^3^-octreotate, the Bcl-2 staining gradually localised to the tumour cell nuclei, and in regrown tumours, it reappeared in the cytoplasm (Figure 4).

### Effect of treatment with^177^Lu-DOTA-Tyr^3^-octreotate

**Figure 5:**
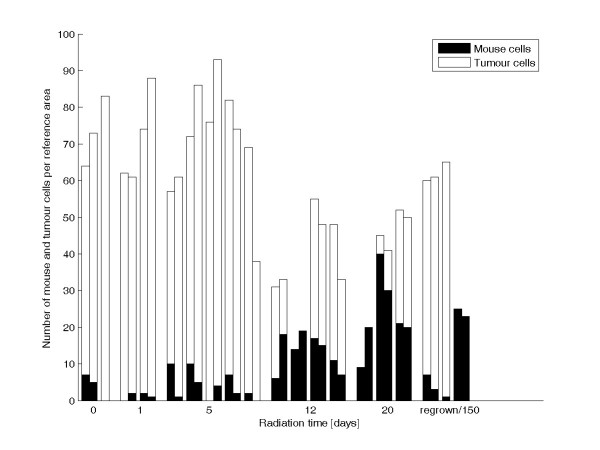
**Radiation effects on densities of mouse and tumour cells**. The *X*-axis shows the numbers of days of^177^Lu-DOTA-Tyr^3^-octreotate treatment. The numbers of mouse and tumour cells were determined for a defined reference area in the centre of the tumour. Bars that are close together represent results from different sections of the same tumour. The numbers of tumour and mouse cells represent different tumours for the untreated group (*n *= 2), the 1-day-treatment group (*n *= 2), the 5-day-treatment group (*n *= 5), the 12-day-treatment group (*n *= 4), the 20-day-treatment group (*n *= 3), and the 150-day-treatment or regrown tumours group (*n *= 3).

The histological examination demonstrated that^177^Lu-DOTA-Tyr^3^-octreotate had a dramatic effect on the tumour cells. After only 12 days of treatment, a 50% reduction in tumour cell density was observed in three of four tumours, and the fourth tumour showed only a few viable tumour cells at the edge of the tumour (Figure 5). After 20 days of treatment, the tumour cell density was further reduced, and in one section, all cells were Ki67 negative and displayed no SCLC cell morphology (interpreted as no detectable tumour cells; Figure 3).

**Figure 6:**
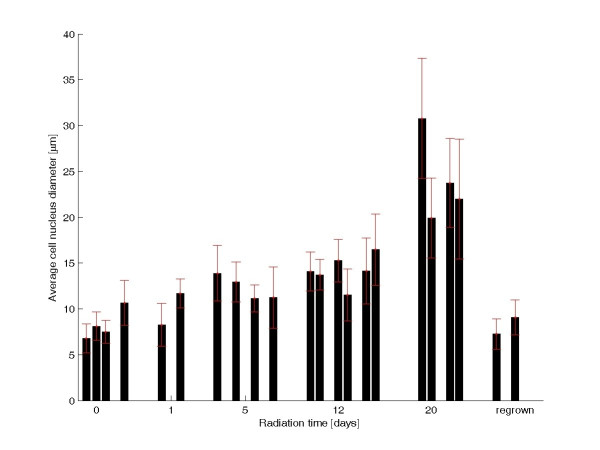
**Radiation effects on tumour cell nuclear diameters**. The *X*-axis shows the numbers of days of treatment. The nuclear diameters were measured in a defined reference area in the centre of the tumour; one to three sections were measured in two to four tumours from each group. Bars that are close together represent results from different sections of the same tumour. The nuclei were measured in different tumours for the untreated group (*n *= 2), the 1-day-treatment group (*n *= 2), the 5-day-treatment group (*n *= 4), the 12-day-treatment group (*n *= 4), and the group with regrown tumour cells (*n *= 2).

The treatment also caused an increase in the average size of the cell nucleus (Figure 1, 3, 4, and 6). After 20 days of treatment, the average nucleus size was nearly three times larger than that of untreated controls. Several of the tumour cells were multinucleated, and some had characteristic micronuclei, where a nuclear membrane surrounded chromosomal material close to, but outside the large nucleus (Figure 1). This multinuclear and/or micronuclear appearance increased with time after treatment.

As mentioned above, histological results showed that the xenograft tumours stained positive for anti-SSTR2 with some unspecific binding. No increase in SSTR2 positivity could be observed in tumour xenograft sections at any time after treatment with^177^Lu-DOTA-Tyr^3^-octreotate.

Untreated controls and regrown tumours were similar in TS1, anti-Ki67, and anti-Bcl-2 staining. They also showed similar cell numbers and nuclear sizes.

**Figure 7:**
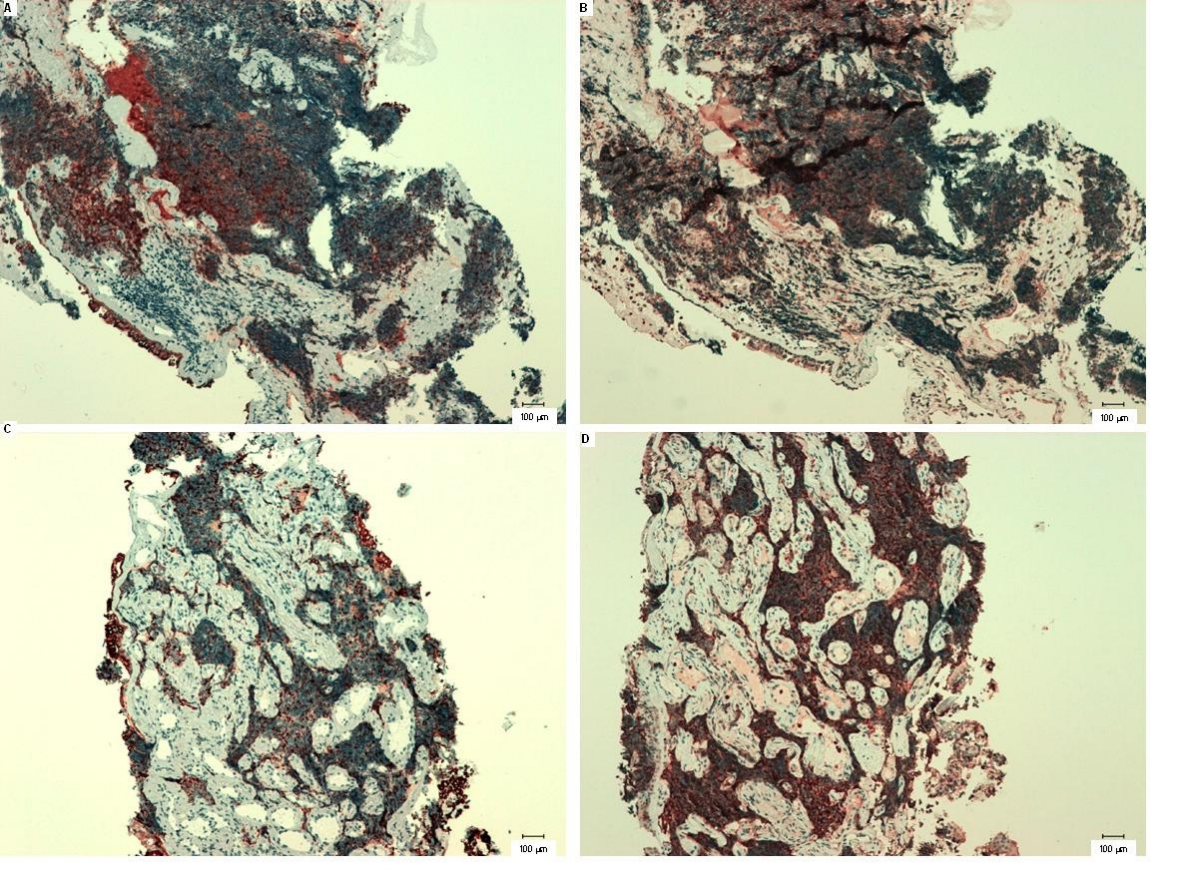
**Serial sections of primary tumours from humans with small-cell carcinoma in pulmonary bronchi**. (**A**, **B**) representative serial sections from patient 1; (**C**, **D**) representative serial sections from patient 2. (A) and (C) show sections labelled with the anti-K8 antibody, TS1; (B) and (D) show sections labelled with an anti-SSTR2 antibody.

**Figure 8:**
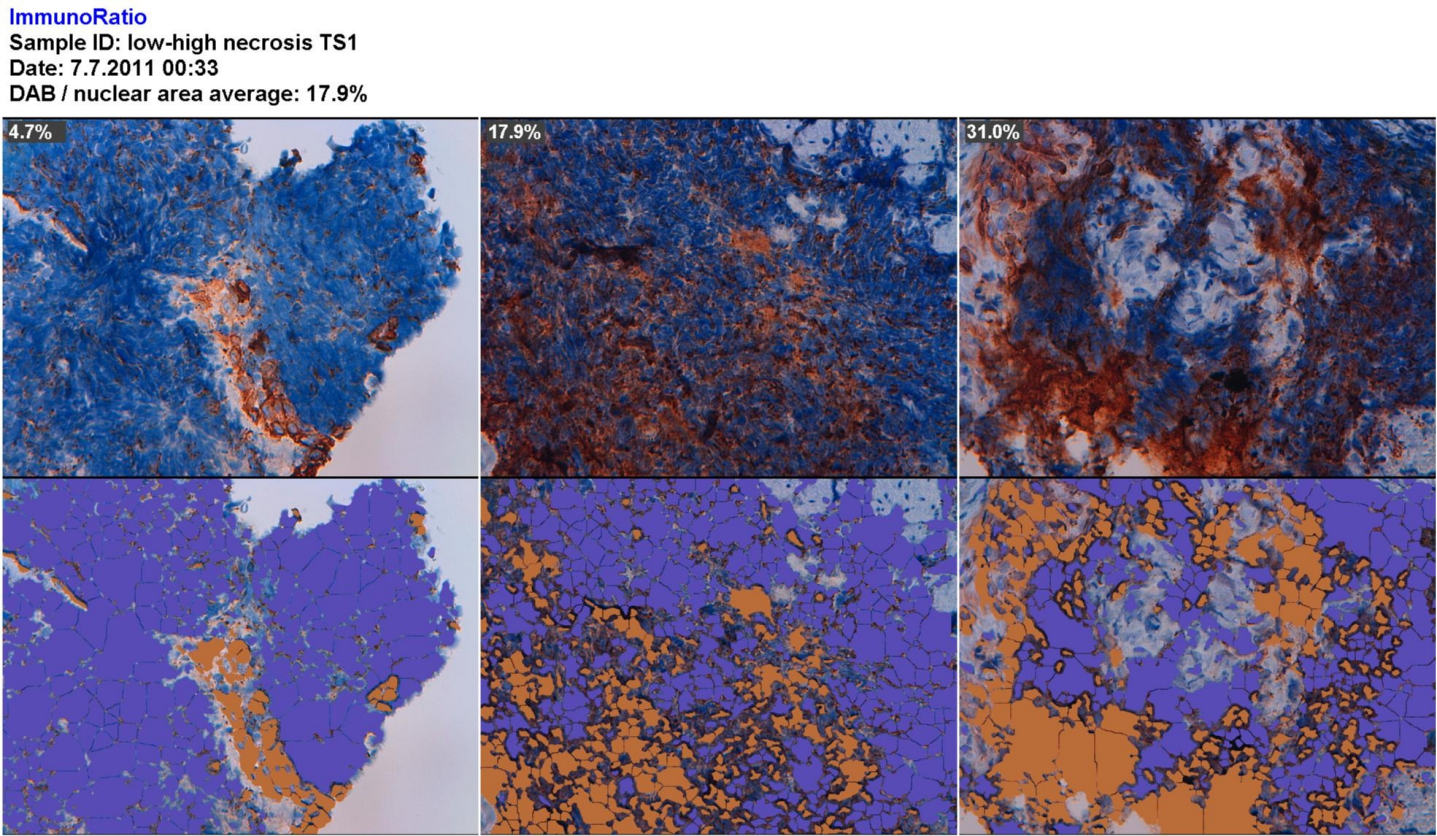
**ImageJ ImmunoRatio result from the analysis of the primary bronchial small-cell carcinomas**. One case with low staining (case 4, left), one case with medium staining (case 1, middle) and one case with high staining (case 5, right).

Serial sections of specimens from seven humans with primary bronchial small-cell carcinoma were also studied with the TS1 and anti-SSTR2 antibodies. In one of the two cases analysed with both antibodies (case 1; Figure 7), we observed similar binding patterns for TS1 and anti-SSTR2. In the other case (case 2; Figure 7), the staining for SSTR2 was more intense. The biopsies had approximately the same size, 10-20 mm^2^, but the number of areas with tumour cells, necrotic tissue, and high TS1 staining varied. Between two to nine 320 × 250-μm areas with tumour cells, necrotic tissue, and high TS1 binding per case were analysed with ImageJ ImmunoRatio to determine the DAB staining after TS1 immunohistochemistry (Table 2). ImageJ ImmunoRatio results from three cases, with low, medium, and high staining, are presented in Figure 8.

**Table 2:** Level of TS1 staining in selected regions of primary bronchial small-cell carcinoma biopsies

Case	Image 1	Image 2	Image 3	Image 4	Image 5	Image 6	Image 7	Image 8	Image 9
1	17.9	45.2	44.1	12.3	8.6	41.8	31.6	-	-
2	44.9	19.0	16.1	-	-	-	-	-	-
3	8.0	5.5	-	-	-	-	-	-	-
4	4.7	5.1	-	-	-	-	-	-	-
5	31.0	22.9	430	32.9	68.7	58.9	71.0	60.8	49.5
6	9.9	15.7	3.3	4.4	-	-	-	-	-
7	81.7^a^	11.6	9.0	11.2	13.1	14.4	-	-	-

The biopsies had approximately the same size 10-20 mm^2 ^but the number of areas with tumour cells and necrotic tissue varied. Between two to nine 320 × 250-μm areas with tumour cells, necrotic tissue, and high TS1 binding per case were analysed. The percentage (%) DAB-positive immunostaining for TS1 in seven cases of primary bronchial small-cell carcinomas was determined by use of ImageJ ImmunoRatio.^a^Contains many intact tumour cells.

## Discussion

Our results showed that treatment with three fractions of 30 MBq^177^Lu-DOTA-Tyr^3^-octreotate given in 24-h intervals had pronounced effects. The activity per fraction was restricted to 30 MBq per injection, because higher amounts had demonstrated saturation [27]. However, the cumulative activity was 90 MBq, which was equal to that measured in our previous study with two treatments of 45 MBq [18]. This new fractionation protocol appeared to be more effective, although this observation was not statistically significant, probably due to the limited number of animals included in both studies.

The goal in this study was to achieve a high tumour response and to analyse the histological changes that occurred during therapy. Our focus was on K8 expression during therapy. To strengthen the claim of treatment efficacy based on observed increases in K8 expression during therapy, we included mice xenografted with the human midgut carcinoid cell line, GOT1.

For the SCLC cell line H69 tumours, the three times 30 MBq protocol caused minor effects after 1 day, somewhat more pronounced after 5 days, and extensive after 12 and 20 days. After 12 days, we observed a nearly 100% regression (cell number) in one of four tumours (*n *= 4); in the other three specimens, the cell number had reduced to about 50% of that observed in the untreated control. After 20 days of treatment, tumour cells were eliminated in one of three tumours (*n *= 3). In the other two tumours, the cell density had reduced to one fourth of that observed in the untreated control. It is well known that irradiation of tumour cells can induce several cell-death mechanisms, including apoptosis, necrosis, autophagy, senescence, and mitotic catastrophe [41]. A striking histological finding was the enlarged nuclei observed in sections from tumours at 20 days after^177^Lu-DOTA-Tyr^3^-octreotate treatment. These nuclei had enlarged to about three times the size of the nuclei in the untreated control. This enlargement was sometimes accompanied by the appearance of multinucleated cells. The formation of multinucleated giant cells was most likely a result of mitotic loss or mitotic catastrophe in the cells.

It has previously been demonstrated that the expression of SSTR2 mRNA was upregulated as a result of^177^Lu-DOTA-Tyr^3^-octreotate treatment. This suggested that fractionated^177^Lu-DOTA-Tyr^3^-octreotate treatment could provide the optimal effect [25,26]. The human and mouse SSTR2 proteins are 98% homologous; thus, it is likely that the monoclonal rabbit anti-SSTR2 antibody used in this study could bind to both human and mouse SSTR2. We observed no increase in SSTR2 staining in the tumour xenograft after treatment. Human and mice have different DNA sequences in the coding regions of the *SSTR2 *gene. According to BLAST alignments, the *Homo sapiens *somatostatin receptor 2 (NM 001050.2) has 88% identity with the *Mus musculus *somatostatin receptor 2 (NM 009217.3). However, the mRNA levels of a gene do not always correlate to the levels of protein expression. Thus, PCR-based methods may provide more accurate information on the expression of mouse and human SSTR2 mRNA in this type of xenograft system.

By visual examination, the positivity of TS1, anti-Ki67, and anti-Bcl-2 appeared to increase in the remaining viable cells of the treated tumours (Figure 1, 3, and 4). There are two subtypes of keratins, the relatively acidic, type I, and the basic, type II, the different types can form complexes in a 1:1 molar ratio a process that leads to the formation of 10-nm-thick filaments [6,33]. In the SCLC cell line H69, the type I keratins 18 (K18), form complexes with K8 of type II. The increase in TS1 binding may have been due to the irradiation of the cells, which may have induced apoptotic caspase-cleavage of K18 and subsequently an increased exposure of the keratin 8 epitope [42]. However, relatively few apoptotic cells were observed in the sections. Alternatively, non-apoptotic mechanisms that induced phosphorylation or other modifications of keratin 8, which subsequently rearranged the cytoskeleton, may have led to increased exposure of the TS1-related keratin 8 epitope [43]. The histological sections also showed that Bcl-2 accumulated in the nuclei after radiation exposure (Figure 4). It was previously demonstrated that Bcl-2 could inhibit apoptosis and also inhibit multiple DNA-repair pathways; thus, tumour cells exposed to DNA-damaging substances, like nitrosamine, exhibited an accumulation of Bcl-2 in the nuclei [44]. The high expression of Ki67 and Bcl-2 in combination with the non-functional p53 could have contributed to cell tumourigenicity, regrowth of tumour cells, and the formation of radiation-damaged multinucleated giant cells with improper chromosome segregation and cell division behaviours [41,45,46]. The majority of these giant cells had probably divided a few times to become polyploidy/multinucleated cells that eventually died by delayed apoptosis or necrosis [47,48]. However, some theories have suggested that these cells may survive through a reverse polyploidy pathway that may be activated by the self-renewal stem cell genes, NANOG, OCT4, and SOX2 [49]. Despite the extensive effects of^177^Lu-DOTA-Tyr^3^-octreotate treatment observed after 12 and 20 days, only one of four tumours showed 100% regression after 150 days; the recurrent tumours were histologically similar to untreated tumours. This suggested that, in the majority of tumours, a few cells must have escaped radiation treatment and regained tumour cell properties and viability. Resistance to treatment, relapse, and the presence of metastases has been attributed to the presence of resting cancer stem cells. Other studies have identified cells with the stem cell marker, CD-133, in SCLC tumours [8]. However, we did not find any CD-133 positive cells in sections from the H69 SCLC xenografts.

Viable tumour cells could be detected within in the outermost regions of large necrotic areas in untreated (Figure 1C), 1- and 5-day-treated tumours as well as regrown tumours. It is important to reach and kill these few viable cells that have escaped the previous treatment. However, resting viable tumour cells within necrotic areas are usually difficult to reach with immuno-targeting agents not only due to the lack of blood supply but also because of absent or low antigen concentration. The biochemical properties of K8, including low solubility, high stability, and intracellular expression, differ from those of membrane-bound target antigens. If the target antigen is present in very high concentrations close to the tumour cells, like K8 in these necrotic regions, targeting is expected to be more successful compared to a membrane-bound antigen. To our knowledge, targeting exposed intracellular tumour antigens is a unique approach. Currently, TS1 is the only antibody that has been shown, in experimental studies, to kill tumour cells by targeting an intracellular antigen exposed in necrotic tissue [30,31,50,51].

We detected an increased exposure of the TS1 target, K8 in particular in small necrotic areas in tumours after 5 and 3 days of^177^Lu-DOTA-Tyr^3^-octreotate treatment in both the NCI-H69 SCLC and midgut GOT1 xenografts. The optimal time point to administrate TS1 should be when the level of smaller necrotic regions is high. *In vivo *diffusion capability of TS1 have been demonstrated earlier [30,31,50,51] and these small areas of necrotic tissue created by^177^Lu-DOTA-Tyr^3^-octreotate treatment as well as the outermost areas of larger necrotic regions can probably be reached by diffusion of the TS1 antibody over short distances *in vivo*.

High affinity interaction between an antibody and its antigen not only enables a longer antibody retention in tumours it can also reduce the distance an antibody will diffuse within a tumour, therefore comparison of intact and fragments of TS1 should be studied in the future. Recently, monovalent and divalent scFv forms of TS1 were generated that had better diffusion capabilities [52]. It was also demonstrated that a combination of the TS1-binding, anti-idiotypic antibody, αTS1, in complex with TS1 provided improved tumour penetration in HeLa HEp-2 multicellular tumour spheroids and experimental tumours [51].

In the human biopsies of primary bronchial small-cell carcinoma, tumour cells showed high positivity for SSTR2 and normal cells tended to be negative. In contrast, these biopsies showed positivity for TS1 in normal lung epithelial cells, tumour cells, and in necrotic regions between fast growing viable tumour cells. *In vivo*, one would not expect TS1 to target intact viable normal cells or tumour cells. However, necrotic regions present in the biopsies were generally small, and close to fast dividing tumour cells. These facts might facilitate contact between TS1 and K8 and increase the distribution of TS1 close to viable tumour cells *in vivo*. Image analysis of the biopsies demonstrated that five out of seven patient biopsies in the analysed regions expressed K8 to an equal or higher level than that observed in untreated SCLC xenografts. In addition, a pre-radiation treatment with^177^Lu-DOTA-Tyr^3^-octreotate would most likely further increase the level of exposed K8 in the tumour.

## Conclusions

There is an urgent need for new improved treatment modalities for SCLC. The results presented in this study are relevant to the understanding of potential pathways for development within this field. Our results on the tumour xenografts suggested that^177^Lu-DOTA-Tyr^3^-octreotate, when administered in optimised, fractionated doses in combination with other treatments, could be an alternative treatment also for SCLC.

Our results demonstrated that^177^Lu-DOTA-Tyr^3^-octreotate treatment increased the TS1 accumulation *in vitro *in SCLC and midgut xenografts. This indicated that the combination of^177^Lu-DOTA-Tyr^3^-octreotate and radiolabelled TS1 could be valuable to investigated further since it holds promise as a future treatment for SCLC.

## Abbreviations

ER: endoplasmic reticulum; K18: keratin 18; K8: keratin 8; SCLC: small-cell lung carcinoma; SSTR2: somatostatin receptor subtype 2

## Competing interests

The authors declare that they have no competing interests.

## Authors' contributions

AE and PB initiated the idea to use TS1 in the SCLC and GOT1 xenografts. AE performed the immunohistochemistry and the interpretation of the histological results, calculation of mouse and tumour cells and drafted the manuscript. PB and EFA conceived the animal study, and helped to draft the manuscript. TS performed the study with biopsies from primary small-cell lung carcinoma and was involved in the interpretation of the histological result as well as reading and approval of the final manuscript.
